# Translational research and key aspects to make it successful

**DOI:** 10.1186/s41231-023-00153-9

**Published:** 2023-09-18

**Authors:** Animesh Acharjee

**Affiliations:** 1 Institute of Translational Medicine, University Hospitals Birmingham NHS Foundation Trust, Birmingham, UK; 2 Institute of Cancer and Genomic Sciences, College of Medical and Dental Sciences, University of Birmingham, Birmingham, B15 2TT UK; 3 MRC Health Data Research UK (HDR UK), London, UK; 4 Centre for Health Data Research, University of Birmingham, Birmingham, B15 2TT UK

**Keywords:** Translational research, Leadership, Project management, Communication

Translational research [1] refers to the translation of scientific discoveries into practical applications that can benefit patients and the wider society. In translational research, basic scientists and clinicians collaborate to develop research questions and plan for testing, implementing new interventions for the bed applications. So, in a way translational research [1] aims to improve human health through the integration of basic science and clinical practice. However, it requires special skillsets that are less pronounce in traditional clinical or basic research.

This letter aims to identify the key soft skills parameters that are essential for successful translational projects.

## Academic leadership vs. transformative leadership

Leadership can be defined as the ability to motivate, inspire, and direct people or groups towards a common goal. In translational research, leadership can include overseeing a team consisting of healthcare professionals. It also involves setting priorities and objectives for the organisation [2]. It is vital to implement policies and strategies that improve education quality, support faculty, staff, and uphold institution's mission and objectives. In several ways, translational leadership is different from academic leadership: Translational research leadership must be more patient-centric and people-cantered. It should also focus on the integration of individual expertise into a larger framework. Facilitate interactions between clinically motivated issues and non-clinical elements such as statistical or mathematical considerations. These skills can be developed while maintaining cultural humility. This will result in stronger translational research teams and increased satisfaction at the locations where they are applied.

## Management of the translational projects

Project management is a method of ensuring that complex tasks are completed on time and in a systematic manner. This involves applying relevant data, tools, and skills in a logical, structured, and efficient way. Project management is essential for translational research and it requires collaboration and mainly coordination between many departments. It can be very beneficial when specialists with different skills or professions collaborate on specific tasks. Although project management is well-established in many other industries, it has been less popular in academic science and clinical research. In the past two decades, there has been a rise in funding for collaborative research projects that bring together subject matter experts from different fields to solve scientific problems. The federal Programme Management Improvement and Accountability Act (Federal Programme Management Improvement and Accountability Act) further demonstrated this trend in this domain.

## Communication

Communication is the key in the translational research as it improves everyone's awareness and keeps them informed about the situation’s arounds the projects. One of the examples would be in the area of the translational diagnostics research where discovery is made by computational or quantitative group and trial performed by another group. Those groups need to be constant communication on the updates and follow ups needed accordingly. One of the best ways to communicate and getting periodic update using an organised meeting with an agenda. Such meetings helps to keep everything in order and ensures that all topics are covered. In addition to this, brief report or minutes document helps to make decisions and prioritize actions and hold participants accountable for their responsibilities. Thus, it provides an unique opportunity to bridge the gap in translational medicine [3].

## Team composition and dynamics

For teams to achieve their translational driven goals, team dynamics [4] is essential. It creates an environment that encourages, produces work, and helps its members grow professionally. The interactions, relationships, communication patterns, and performance of team members can have an impact on their overall effectiveness and performance. Team dynamics that work well emphasize cooperation, mutual respect and open communication. They also encourage inclusion with people who are supportive. Different perspectives, backgrounds and experiences can benefit teams for example: inclusion of the machine learning and clinical expert in the same team. However, it can also lead to conflicts which must be managed well using soft skills like conflict or stress management. Team members need to have faith in each other's abilities, intentions, as well as their dedication. Team success is dependent on the ability to resolve conflicts and hence it is important to take timely steps, understand each other's perspective, and find mutually beneficial solutions.

## Collaboration and network

Collaboration is the key in this domain. Researchers in the lab-based projects or clinic are expected to combine the expertise and work in a collaborative environment. This also helps communities to figure out what kind of health innovations they need [5]. Eventually, those collaborations help us to make a network of people with multiple expertise and impact on the society. Most of the time, translational research results can be used and influence society. This is generally done at one of three stages: initial research to influence, research that applies to society, or research on society. However, the important question is how can we get the next generation leaders interested in the translational research that can help people? Networks and the interactions may be the first step towards it.

## Roles and responsibilities

Translational research is an interdisciplinary domain that combines scientific discoveries with practical applications in healthcare. Hence, it involves various roles and responsibilities, including basic researchers conducting fundamental research, clinical researchers conducting clinical trials. Translational scientists serving as a bridge between basic researchers and clinicians. Each role plays a vital role in bringing scientific discoveries to the forefront of patient care and public health. Each of the roles need to be defined properly with some flexibility to adapt and move forward. In case of more dynamic roles, a training and integration programme need to be designed.

## Conclusions

**Fig. 1:**
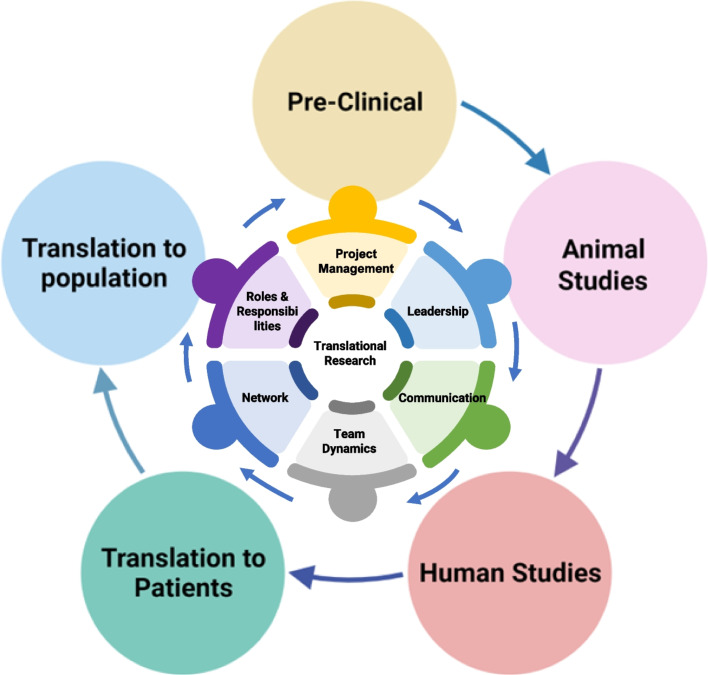
Multiple processes and steps around translational research is shown. The outer periphery is more on the translational processes and inner circles are more on the soft skills that require to execute the processes in the outer circles

It is worth taking the time to realise the complex nature of the translational research and their multiple components. We often focus on the outer circle (Fig. 1) which is technology or domain specific whereas inner circle which is mainly focused on the non-technical skills are also important. Over the years, translational research has traditionally followed the path of the technology outer circle in Fig. 1, this has presented challenges because brilliant academic research has rarely translated effectively primarily because it is not directed at clinically critical questions as opposed to good science, as a result, the bench-to-bedside model, as outlined in this work, is evolving to recognize that it must be bed-to-bench-to-bed. To be successful in this area, a training programme in translational research must provide its trainees with exposure to and practise in a wide range of abilities that are typically not covered in a single curriculum. We, as a scientific community, need to welcome and develop multidisciplinary teams from across institutions into our labs, where they may be recognized and rewarded for taking on the difficult task of finding answers rather than raising additional questions. There is a long road ahead for the next generation of researchers who may not follow the standard path to success in academia, and it is imperative that we, as administrators, teachers, and mentors, continue to invest in them.

## Data Availability

Not applicable.
